# Protective role of summer acupoint application therapy on weather-triggered rheumatoid arthritis flares

**DOI:** 10.1016/j.onehlt.2026.101574

**Published:** 2026-09-14

**Authors:** Ling Xin, Jian Liu, Wei Song, Yang Li, Yongjian Zhu

**Affiliations:** a The First Affiliated Hospital of Anhui University of Chinese Medicine, Hefei, China; b School of Government, Nanjing University, Nanjing, China

**Keywords:** Diurnal temperature range, Rheumatoid arthritis, Summer acupoint application, Proactive health, Case-crossover design

## Abstract

Summer acupoint application (SAA) therapy is widely practiced in China for rheumatoid arthritis (RA) prevention, but its effectiveness in mitigating weather-related RA risk remains unclear. This study evaluated the short-term association between diurnal temperature range (DTR) and RA hospitalization, and quantified SAA's protective effect using daily hospital data from Hefei, China (2013–2024; *n* = 9261, including 1066 SAA recipients). A time-stratified case-crossover design combined with 1:1 propensity score matching (PSM) was employed. Per interquartile range increase in DTR, cumulative RA hospitalization risk rose by 10.2% (95% CI: 2.7%–18.2%) at lag 0–7 days. After PSM, the control group exhibited elevated risk (OR = 1.138, 95% CI: 1.013–1.279), whereas the SAA group showed no significant DTR-related risk (OR = 0.889, 95% CI: 0.734–1.075), with a significant between-group difference (*P* = 0.030). Attributable fractions were 12.13% and − 12.54% for control and SAA groups, respectively. The protective effect of SAA therapy was stronger among females, patients aged ≥45 years, and those with more comorbidities, and was most pronounced in autumn. These findings indicate that SAA therapy may mitigate DTR-related RA hospitalization risk, highlighting a potential preventive strategy for vulnerable populations. What is innovative in this paper is that the linking of meteorological stressors with a traditional Chinese medicine (TCM) intervention was first explored, and the results of the study also showed that TCM intervention was indeed associated with a reduced risk of DTR-related RA hospitalizations. However, the observational nature of this study precludes causal inference, and future randomized controlled trials should be employed to validate these findings further.

## 1 Introduction

Diurnal temperature range (DTR), defined as the difference between the daily maximum and minimum temperatures, which captures the sudden within-day temperature variation, is a well-recognized threat to global public health. A growing body of evidence indicates that DTR increases the morbidity and mortality of numerous diseases [40]. Sudden within-day temperature changes are closely associated with the development of inflammation [24], which plays a critical role in the onset or exacerbation of autoimmune diseases such as rheumatoid arthritis (RA) (J. [19]). RA is a chronic, systemic autoimmune disease characterized by persistent synovial inflammation and progressive joint destruction, which leads to severe disability, impaired quality of life, and a considerable socioeconomic burden worldwide [30]. Given that disability-adjusted life-years attributable to RA have nearly doubled over the past 31 years [18], the association between DTR and RA warrants widespread attention. A recent systematic review and meta-analysis confirmed that weather conditions are significantly associated with RA disease activity [12], and a prior study conducted in Hefei reported substantial short-term effects of DTR on RA hospital admission risk (J. [27]). Nevertheless, most previous studies have focused on describing these exposure–risk associations, and it remains largely unknown whether weather-related RA exacerbations can be effectively prevented.

The long-term conventional treatment of RA (including disease-modifying anti-rheumatic drugs, nonsteroidal anti-inflammatory drugs, etc.) may cause liver and kidney damage, which prompts researchers to explore alternative therapies for RA with fewer side effects [4]. Summer acupoint application (SAA) therapy is a proactive health intervention in which herbal medicinal paste is applied to selected acupoints during the dog days of summer (J. [21]). According to traditional Chinese medicine (TCM) theory, the skin surface and blood vessels are most active during the dog days owing to the highest ambient temperatures and yang qi. SAA therapy thus facilitates the penetration of herbal ingredients through the skin to reach affected areas, thereby dispelling wind, eliminating dampness, and regulating immunity [13]. Accordingly, this treatment is known as “Treating Winter Disease in Summer” and is believed to help prevent disease exacerbation caused by sudden temperature changes during the cold season [13], [33]. As an external, low-cost therapy with fewer adverse effects, SAA has been widely adopted in TCM hospitals and community health centers in China, making it a highly scalable candidate preventive strategy [14].

Numerous studies in China have described the therapeutic effects of SAA, primarily for respiratory diseases, yet its protective effect on RA patients exposed to meteorological stressors remains unexplored. We employed a time-stratified case-crossover design combined with 1:1 propensity score matching (PSM) to evaluate whether SAA therapy can modify RA hospitalization risk due to the DTR stressor. Specifically, the case-crossover design uses each case as its own control, thereby inherently controlling for unmeasured individual-level confounders such as lifestyle and socioeconomic status [36], whereas PSM can be used to balance measured baseline characteristics between the SAA-treated and control groups, approximating randomized treatment assignment (D. [31]). Importantly, to the best of our knowledge, no previous study has employed this methodological combination to assess the association between TCM intervention and weather-related health risk; this method therefore constitutes a key novelty of the present work. Moreover, although the adverse effects of meteorological factors on RA have been reported, few studies have proposed effective interventions to reduce weather-related RA risk.

Accordingly, this study aimed to determine whether SAA therapy can protect RA patients against weather-triggered flares. Using 12 years of daily hospital data from Hefei, China, we pursued two specific objectives: (1) to quantify the short-term association between DTR exposure and RA hospitalization and (2) to evaluate whether SAA therapy mitigates DTR-related RA hospitalization risk.

## 2 Materials and methods

### 2.1 Study area

**Fig. 1:**
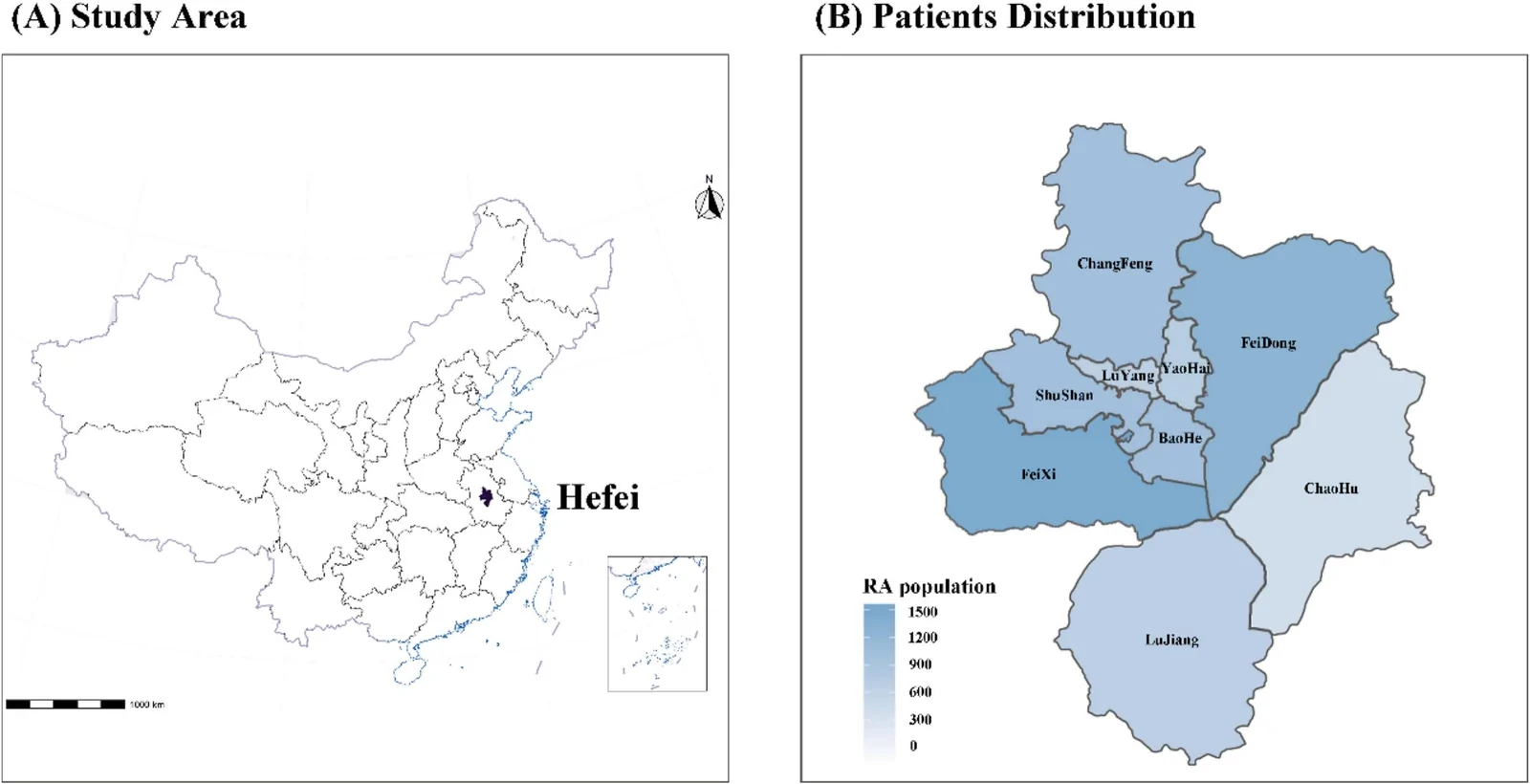
Study location of Hefei in China, and geographical distribution of Rheumatoid arthritis patients in Hefei, China, 2013–2024.

This study was conducted at two tertiary TCM hospitals (The First Affiliated Hospital of Anhui University of Chinese Medicine and Anhui Integrated Traditional Chinese and Western Medicine Hospital) in Hefei, China (Fig. 1). Hefei, the capital of Anhui Province (31°52′N, 117°17′E), covers an area of 11,445 km^2^ and had a permanent population exceeding 10 million in 2024. It is located between the Yangtze River and the Huaihe River and has a subtropical humid monsoon climate.

### 2.2 RA patient data

We obtained daily outpatient, emergency, and hospitalization records from 2013 to 2024 from the two hospitals, which are government-authorized institutions providing TCM services, including SAA therapy. The dataset contained patient-level information on sex, age, residential address, diagnosis, chronic comorbidities at baseline, follow-up dates, and admission dates. A total of 9377 RA patients were identified before the study period. All patients were residents of Hefei and had designated one of the two hospitals as their primary healthcare provider under the medical insurance scheme. Diagnoses and treatment plans for all patients followed the “Guidelines for Diagnosis and Treatment of Rheumatoid Arthritis Combined with Disease and Syndrome” [16]; as the two hospitals were among the principal drafting units of these guidelines, the diagnostic and treatment protocols remained consistent before and after their release. Patients aged <18 years at the start of follow-up and those lost to follow-up were excluded. In addition, to exclude hospitalizations due to non-RA causes, this study only included hospitalization records with RA as the primary diagnosis. Ultimately, 9261 RA patients and 9009 RA-related hospitalizations were enrolled (Table 1).

**Table 1:** Descriptive statistics of the study population in Hefei, China, 2013–2024.

Characteristic	Before PSM		After PSM	
	N	%	N	%
Patients				
Overall	9261	100%	2132	100%
Group				
SAA group	1066	11.51%	1066	50.00%
Control group	8195	88.49%	1066	50.00%
Gender				
Female	7448	80.42%	1896	88.93%
Male	1813	19.58%	236	11.07%
Age^†^				
< Median	4555	49.18%	1026	48.12%
≥ Median	4706	50.82%	1106	51.88%
Comorbidities				
0	7321	79.05%	1606	75.33%
1	1095	11.82%	280	13.13%
2+	845	9.12%	246	11.54%
Hospitalisations				
Overall	9009	100%	2871	100%
Group				
SAA group	361	4.01%	361	12.57%
Control group	8648	95.99%	2510	87.43%
Seasons^‡^				
Spring	2481	27.54%	787	27.41%
Summer	2335	25.92%	772	26.89%
Autumn	2213	24.56%	693	24.14%
Winter	1980	21.98%	619	21.56%

Abbreviations: PSM: Propensity Score Matching, SAA: Summer acupoint application; ^†^ Median age before PSM: 48 years, Median age after PSM: 45 years; ^‡^ Spring (March to May), Summer (June to August), Autumn (September to November), and Winter (December, January, and February).

### 2.3 Meteorological data

Daily meteorological variables—including daily mean temperature, daily maximum temperature, daily minimum temperature, relative humidity, and wind speed—were obtained from the National Centers for Environmental Information (https://www.ncdc.noaa.gov) and the Goddard Earth Sciences Data and Information Services Center (https://disc.gsfc.nasa.gov). DTR was selected as the primary exposure variable and was calculated as the difference between daily maximum and minimum temperatures.

Due to the high Spearman's correlation coefficients between DTR and relative humidity (Table S1, *r* = −0.641, *P* < 0.01), we chose net effective temperature (NET) instead of mean temperature, relative humidity, and wind speed as the control index. The NET was calculated as follows [11]:

$$\mathrm{NET}=37-\frac{37-\mathrm{TEM}}{0.68-0.0014\mathrm{RHU}+\frac{1}{\left(1.76+1.4{\mathrm{WIN}}^{0.75}\right)}}-0.29\mathrm{TEM}\left(1-0.01\mathrm{RHU}\right)$$

where *TEM* denotes temperature, *RHU* denotes relative humidity, and *WIN* denotes wind speed. *NET* is a comprehensive index incorporating temperature, relative humidity, and wind speed; it is commonly used to represent the actual environmental effect on the human body [29]. Compared with single meteorological variables, NET is more consistent with human thermal perception (P. W. [20]).

### 2.4 SAA therapy protocol

SAA therapy was performed by applying herbal cakes to selected acupoints. The herbal cake was prepared by mixing the hospital preparation Xiaoyu Jiegu powder with ginger juice and honey at a ratio of 2:1:1. Xiaoyu Jiegu powder comprises seven Chinese materia medica: Wujiapi (*Acanthopanax gracilistylus*), Dingxiang (*Eugenia caryophyllata*), Chuanxiong (*Ligusticum chuanxiong*), Biba (*Piper longum*), Shengnanxing (*Arisaema erubescens*), Baizhi (*Angelica dahurica*), and Guipi (*Cinnamomum tamala*). The mixture was cut into 1 cm^3^ cubes and fixed with adhesive plaster on the selected acupoints. The acupoints were Dazhui (GV14, unilateral), Feishu (BL13, bilateral), Pishu (BL20, bilateral), Ganshu (BL18, bilateral), Shenshu (BL23, bilateral), and Mingmen (GV4, unilateral). The herbal cake was retained on the acupoints for 4 h.

The SAA therapy was administered during the dog days of each summer (3 sessions per year). All patients receiving SAA therapy were required to make an appointment in advance. Before each treatment session, well-trained physicians assessed each patient's response to the previous session and reminded them to return for the subsequent session.

### 2.5 Statistical analysis

#### 2.5.1 Time-stratified case-crossover design

A time-stratified case-crossover design was adopted to quantify the short-term association between DTR exposure and RA hospitalizations. This design is widely used to assess associations between environmental hazards and health outcomes because it inherently controls for individual-level confounders that are constant or change slowly over short periods (e.g., age, lifestyle, and socioeconomic status) by using each case as its own control [36]. The case day was defined as the date of RA hospitalization, and control days were the same day of the week in the same month and year.

#### 2.5.2 First-stage analysis

In the first-stage analysis, a conditional logistic regression model combined with a distributed lag nonlinear model (DLNM) was applied to estimate the odds ratios (OR) of RA hospitalization associated with short-term DTR exposure [9]. Following common practice [3], a per-interquartile range (IQR) increase in DTR was used as the exposure increment. Exploratory analyses (Fig. S1) suggested that the adverse effects of DTR on RA patients persisted for approximately one week before gradually subsiding. Therefore, we examined the cumulative association between high DTR and RA hospitalizations at lag 0–7 days. A natural cubic spline function with 3 degrees of freedom (df) for daily DTR and NET was employed to capture potential nonlinear effects, based on the quasi-Akaike Information Criterion. A categorical variable (holiday vs. non-holiday) was included to adjust for the potential influence of statutory holidays.

#### 2.5.3 Second-stage analysis

The second-stage analysis evaluated whether SAA therapy modifies the association between DTR and RA hospitalization risk. First, 1:1 PSM was used to address potential imbalances between the SAA and control groups. PSM approximates randomization and helps control for confounding between exposure and non-exposure groups (D. [31]). The nearest-neighbor matching method was applied, with age, sex, and number of chronic comorbidities at baseline as matching variables. However, despite PSM, the potential residual confounding effects brought by unmeasured variables (e.g., socioeconomic status, diet, physical activity, and medication adherence) cannot be completely ruled out.

Second, DTR-related hospitalization risks in the two groups after PSM were reported as cumulative ORs at lag 0–7 days per IQR increase in DTR, and a *Z*-test was used to compare the SAA and control groups [1]. Because prior evidence indicates that RA patients exhibit relatively stable disease activity and lower morbidity within one year after SAA therapy, we restricted the analysis to hospitalization records occurring within one year after SAA therapy in the SAA group [32]. The post-PSM population was stratified by sex (female or male), age (< median or ≥ median), and number of chronic comorbidities at baseline (0, 1, or ≥ 2) to compare SAA effects across subgroups. Seasonal differences in SAA effects (spring: March–May, summer: June–August, autumn: September–November, winter: December–February) were also examined.

Third, the attributable fraction (AF, %) was employed to estimate the disease burden associated with DTR increase across groups [8]. The 95% empirical confidence interval (CI) of AF was calculated via Monte Carlo simulation.

#### 2.5.4 Sensitivity analyses

A series of sensitivity analyses were conducted to confirm the robustness of the primary findings. First, atmospheric pressure and precipitation were added as additional covariates to control for potential confounding by weather variables. Second, the air pollutant PM2.5 was further included in the model. Third, the degrees of freedom for daily DTR and NET were varied from 3 to 4 and 5. All analyses were performed in R software (version 4.4.1), with two-sided *P* < 0.05 considered statistically significant.

### 2.6 Ethics approval

As all participants were anonymized, and personal identification information and privacy variables were not collected, the informed consent was waived by the Ethics Committee of the First Affiliated Hospital of Anhui University of Chinese Medicine. All experimental protocols were reviewed and approved by the Ethics Committee (ethics approval number: 2025AH-57).

## 3 Results

### 3.1 Descriptive characteristics

Table 1 summarizes the baseline characteristics of the 9261 enrolled patients. The median age was 48 years, and 80.42% were female. As shown in Fig. 1, the spatial distribution of the enrolled patients across Hefei City is illustrated. Of these patients, 1066 (11.51%) had received SAA therapy, and 7321 (79.05%) had no chronic comorbidities. From 2013 to 2024, 4358 patients (47.06%) experienced hospitalization or emergency department visits due to RA exacerbation, contributing to a total of 9009 hospitalization or emergency records.

**Fig. 2:**
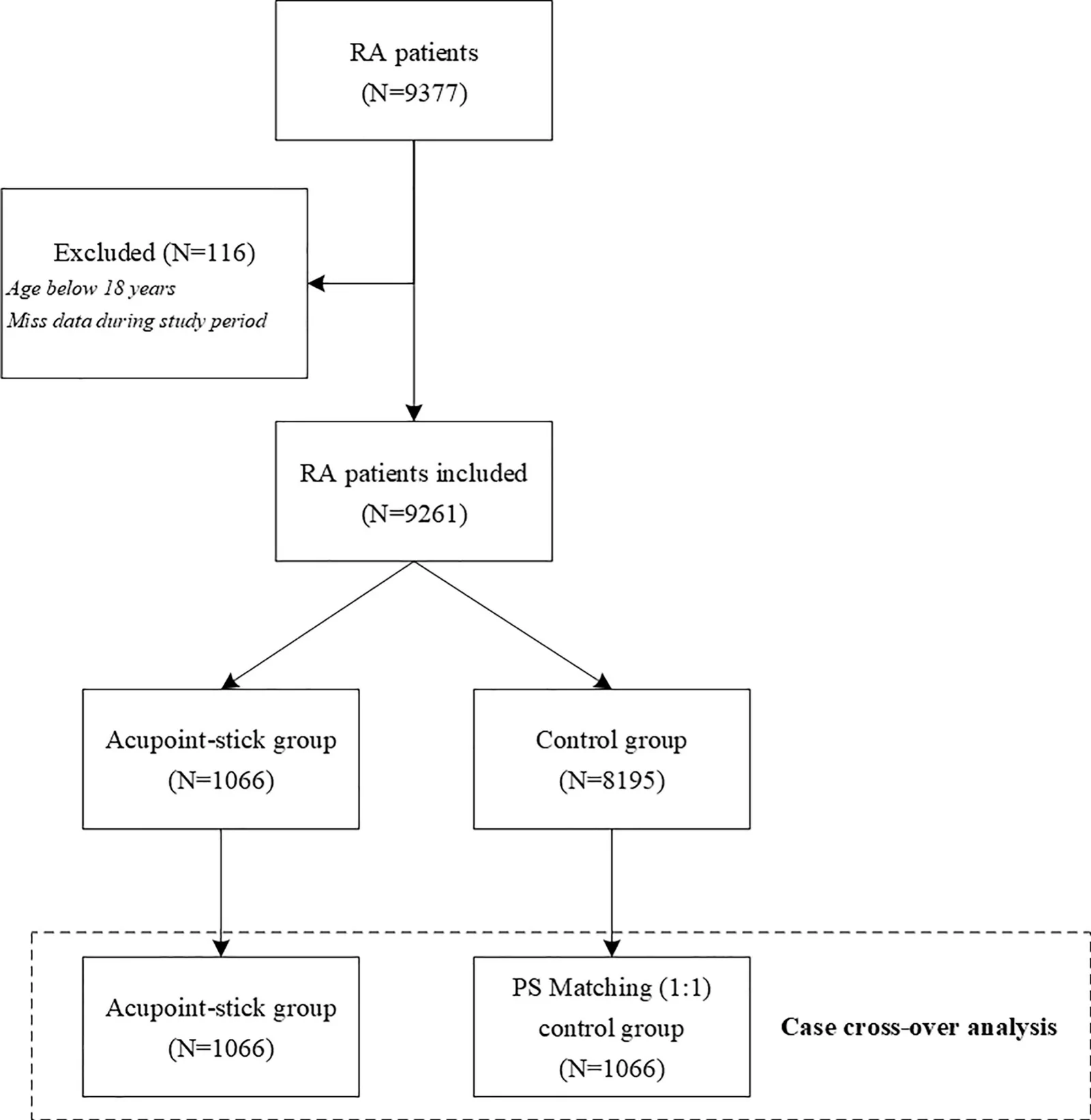
Flow chart for inclusion and exclusion of patients with Rheumatoid arthritis.

To reduce selection bias, 1:1 PSM was applied, yielding 1066 matched pairs for the second-stage analysis, as illustrated in the flowchart in Fig. 2. After matching, no significant differences were observed between the two groups across all matched characteristics (Table S2). Patient characteristics before and after PSM are presented in Tables 1 and S2.

The descriptive statistics and correlations of meteorological variables over the study period are presented in Tables 2 and S1. The daily mean values of DTR, mean temperature, relative humidity, wind speed, and NET were 11.97 °C, 16.83 °C, 68.48%, 5.37 m/s, and 8.21 °C, respectively.

**Table 2:** Description of meteorological variables in Hefei, China, 2013–2024.

Variables				Percentile			
	Mean	SD	Min	P25	P50	P75	Max
Diurnal temperature range (°C)	11.97	4.06	2.12	9.30	11.99	14.82	24.59
Mean temperature (°C)	16.83	9.31	−6.92	8.77	17.63	24.81	34.13
Relative humidity (%)	68.48	14.81	20.92	58.07	69.97	80.03	98.99
Wind speed (m/s)	5.37	1.80	1.16	4.10	5.24	6.49	14.54
Net effective temperature (°C)	8.21	11.86	−22.77	−2.01	9.09	18.46	29.44

### 3.2 Short-term association between DTR and RA hospitalization (pre-PSM)

**Fig. 3:**
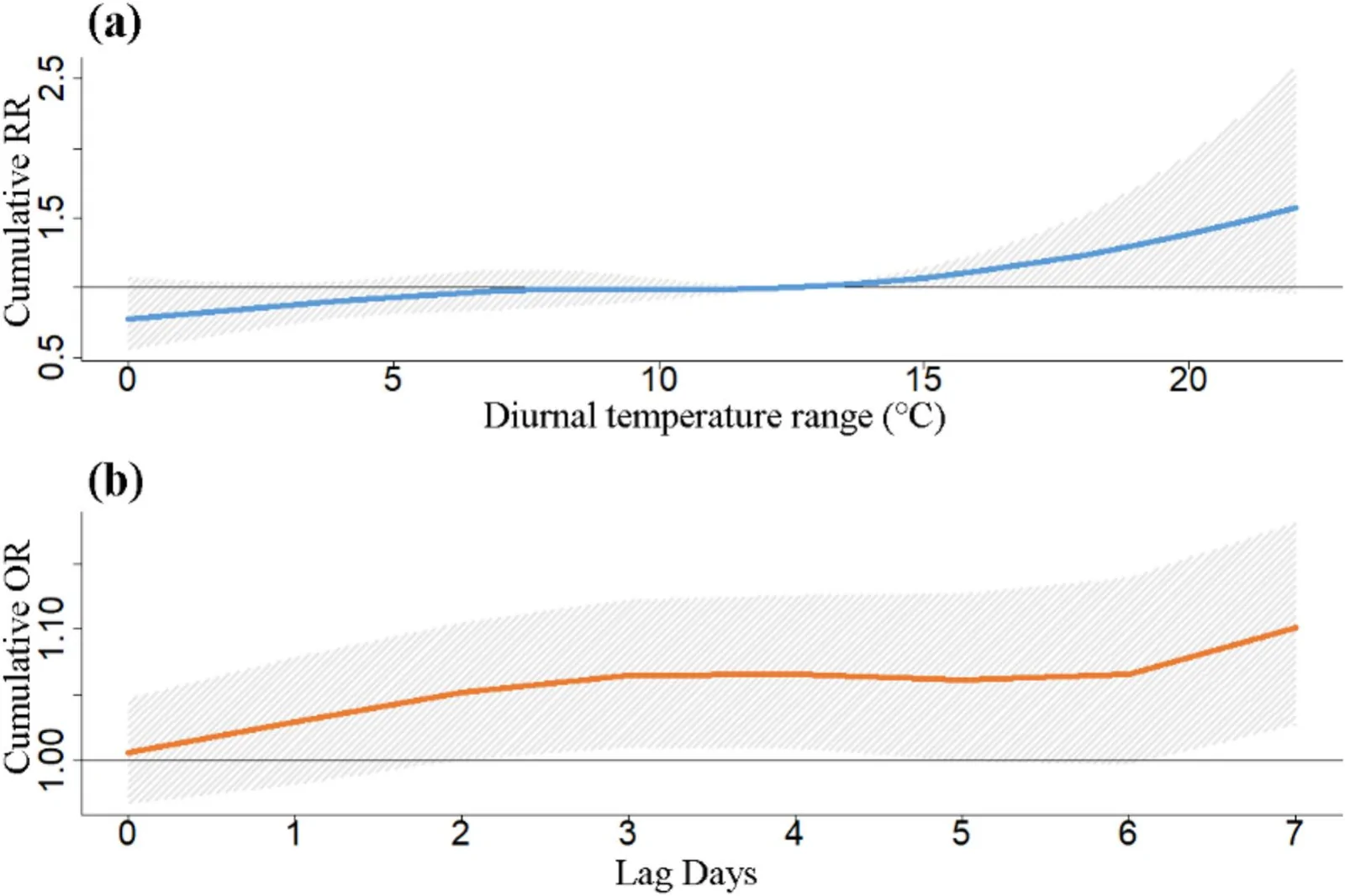
The lag effect structures before PSM of DTR at a lag of 0–7 days on RA hospitalization in Hefei, China, 2013–2024. (a) Overall exposure-response curve for the associations between DTR and hospitalizations for RA, with the comparison to the mean value of DTR. (b) Overall exposure-response curve for the associations between an IQR increase in DTR and hospitalisations for RA. RR: relative risk; OR: odds ratio; CI: confidence interval.

Fig. 3A depicts the cumulative exposure–response relationship at lag 0–7 days between DTR and RA hospitalization risk. The approximately linear curve indicates a significantly elevated hospitalization risk with increasing DTR. Therefore, a linear function was used to model this relationship. Fig. 3B further displays the cumulative effects of per-IQR increase in DTR on RA hospitalization across different lag windows. Specifically, as displayed in Fig. 3B, the significant effect of DTR first emerged at lag 2 days (OR = 1.052, 95% CI: 1.001–1.106) and persisted through lag 7 days (OR = 1.102, 95% CI: 1.027–1.182).

### 3.3 Comparison of DTR impacts between SAA and control groups (post-PSM)

**Fig. 4:**
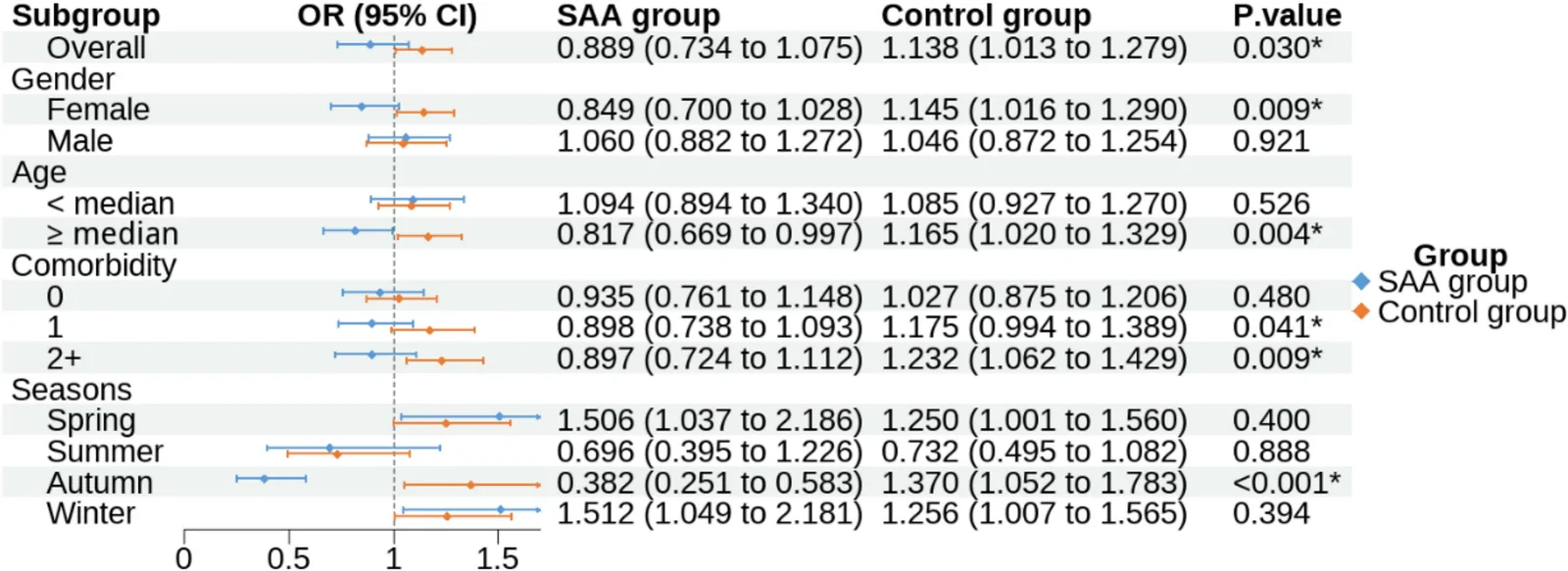
The comparison of DTR–RA hospitalization associations between SAA and Control Groups over 7 lag days.

The comparison of DTR–RA hospitalization associations between the two groups is presented in Fig. 4, where the Overall row shows the estimates for the whole post-PSM population. High DTR was associated with an elevated risk of RA hospitalization in the control group after PSM (OR = 1.138, 95% CI: 1.013–1.279, lag 0–7 days), whereas patients who received SAA therapy showed no significant association (OR = 0.889, 95% CI: 0.734–1.075). The *Z*-test confirmed a significantly weaker association in the SAA group than in the control group (*P* = 0.030), as shown in Fig. 4, indicating that SAA therapy exerts a significant preventive effect on DTR-related hospitalization risk.

As shown in the subgroup- and season-specific rows of Fig. 4, the protective effect of SAA varied by sex, age, comorbidity status, and season. The difference in DTR effects between the SAA and control groups was more pronounced in females than in males (Gender rows in Fig. 4). SAA therapy showed a significant protective effect among patients aged ≥45 years, whereas no significant association was observed in those aged <45 years (Age rows in Fig. 4). In the control group, the odds of DTR-related hospitalization increased with the number of chronic comorbidities; in contrast, hospitalization risk in the SAA group did not vary significantly with comorbidity count (Comorbidity rows in Fig. 4). Additionally, the protective effect of SAA was more prominent in autumn than in other seasons (Seasons rows in Fig. 4).

**Fig. 5:**
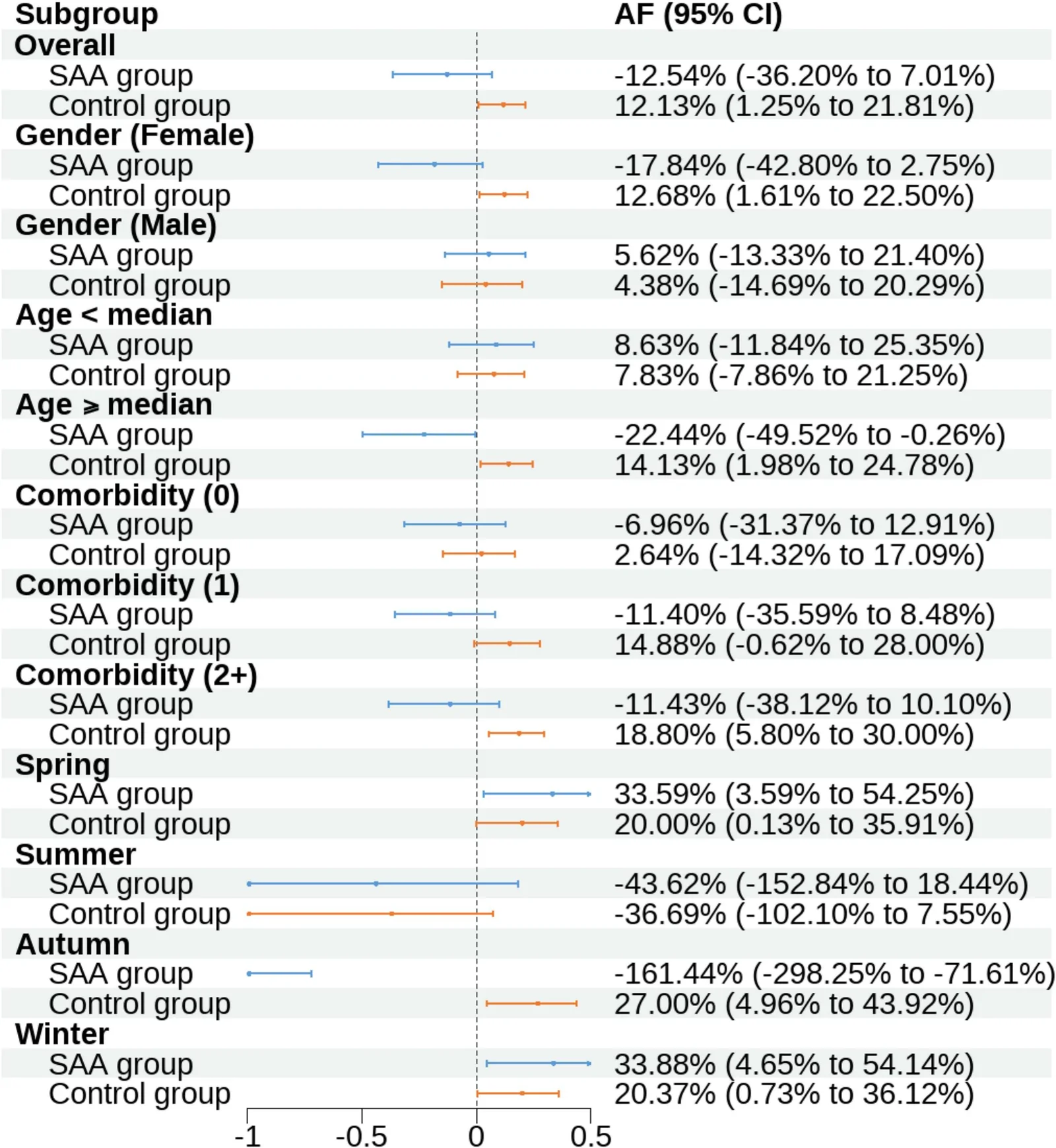
The comparison of DTR-attributable fractions between SAA and Control Groups over 7 lag days.

At the overall participant level, as shown in the Overall row of Fig. 5, the DTR-attributable fraction was higher in the control group than in the SAA group. DTR accounted for 12.13% (95% CI: 1.25%–21.81%) of all hospitalizations in the control group, whereas the AF in the SAA group was −12.54% and not significant. Specifically, 2871 RA hospitalizations were included after PSM (12.57% in the SAA group and 87.43% in the control group; see Table 1), and 304 hospitalizations in the control group were attributable to high DTR. As shown in the subgroup- and season-specific rows of Fig. 5, the DTR-related AF was nonsignificant among SAA-treated patients, whereas DTR yielded substantially higher AF estimates in the control group among three subgroups (female, age ≥ 45 years, and comorbidities ≥2) and during autumn.

### 3.4 Sensitivity analyses

Sensitivity analyses demonstrated that the results were robust. As shown in Figs. S2–S4, the effect estimates changed little after additionally adjusting for atmospheric pressure, precipitation, or PM2.5, respectively. Furthermore, as shown in Figs. S5 and S6, varying the degrees of freedom for meteorological variables from 3 to 4 or 5 did not lead to substantial changes in the results.

## 4 Discussion

### 4.1 Principal findings

In this observational study of RA patients, we identified a significant association between DTR and RA hospitalization risk and, for the first time, explored whether SAA therapy reduces DTR-related hospitalization risk. Our findings demonstrate that each IQR increase (5.62 °C) in DTR was associated with a 10.2% increase (OR = 1.102, 95% CI: 1.027–1.182) in cumulative RA hospitalization risk at lag 0–7 days in the overall pre-PSM population, which imposes a considerable disease burden on the national health insurance system. After PSM, the DTR–RA hospitalization association remained significant in the control group (OR = 1.138, 95% CI: 1.013–1.279), whereas it was no longer detectable in the SAA group (OR = 0.889, 95% CI: 0.734–1.075); the between-group difference was statistically significant (*P* = 0.030), indicating that SAA therapy was associated with a reduced risk of DTR-related hospitalization within one year. Subgroup analyses indicated that the relevance was more pronounced in women and individuals aged ≥45 years, and was stronger in patients with more chronic comorbidities. Moreover, the relevance was predominantly observed in autumn, whereas the difference in DTR-related hospitalization risk between the SAA and control groups was not significant in other seasons. Collectively, these results suggest that SAA therapy, as a representative TCM intervention for RA, has demonstrated promising preventive effects. Promoting SAA therapy may offer a viable strategy for controlling RA incidence and alleviating the burden on national health insurance.

### 4.2 Comparison with previous evidence

This study introduced DTR, a classical meteorological index reflecting acute within-day temperature variation, and observed a significant association with RA hospitalizations independent of NET. The magnitude of this association translates into a considerable attributable burden at the population level, as reflected by the estimated AF of 12.13% (95% CI: 1.25%–21.81%, 304 admissions) in the control group. From a biological perspective, DTR is likely responsible for the short-term effects of low-temperature stimuli on RA patients, as the human body typically requires a stress-regulated adaptive process following acute environmental stressor exposure [2]. This finding is consistent with previous studies linking temperature decreases and cold spells to increased RA hospital admission risk [12]. However, most prior studies have focused on the epidemiological associations between meteorological factors and disease risk [37], and effective risk-reduction interventions remain to be developed. This gap underscores the urgent need for effective proactive health strategies to enhance the self-protection capacity of RA patients against weather changes (J. [21]). SAA, as a representative proactive health intervention, may help fill this gap.

Previous clinical observations in China have reported that SAA can improve the quality of life and perceived treatment efficacy of patients, primarily those with respiratory diseases such as asthma, rhinitis, and chronic bronchitis [13]. However, to our knowledge, no prior study has examined the modifying effect of SAA on the acute association between sudden weather changes and RA episodes, making direct comparison with previous studies challenging. The pronounced difference in DTR-related RA hospitalization risk between the SAA and control groups observed in the present study (OR = 1.138 vs. 0.889, corresponding to approximately 28% higher odds in the control group; *P* = 0.030) therefore provides novel evidence for a potential protective effect of SAA on RA patients. Although this between-group difference was only marginally significant, its consistency with the substantially different AF estimates of the two groups supports a genuine protective effect.

### 4.3 Potential mechanisms

The mechanisms by which DTR triggers acute RA episodes are not fully understood; however, studies on immune function inhibition and inflammatory responses provide plausible explanations [34]. High DTR may disrupt the body's temperature rhythm and induce thermoregulatory fatigue, potentially suppressing immune function and increasing the risk of acute RA episodes [22]. Moreover, sudden temperature changes may provoke joint pain in RA patients by altering C-reactive protein levels and triggering inflammatory responses [10].

Immunomodulation and anti-inflammatory effects are recognized as key therapeutic targets in RA management [30]. These effects may help the body counteract DTR stress; accordingly, the pharmacological properties of the herbal cake used in SAA may contribute to the observed protective effect. From an immunoregulatory perspective, the active ingredients of the herbal cake, delivered via transdermal permeation, may help restore immune balance: polysaccharides from Acanthopanax gracilistylus (Wujiapi) promote lymphocyte proliferation and upregulate multiple cytokines [14], and eugenol, the major active component of clove (Dingxiang), inhibits dendritic cell maturation and decreases TNF-α, interferon-γ, and monocyte infiltration in the collagen-induced arthritis (CIA) model [15].

From an anti-inflammatory perspective, components of the herbal cake directly suppress inflammatory pathways: lignans and caffeoylquinic acid derivatives from Wujiapi selectively inhibit cyclooxygenase-2 (COX-2) and neutrophil elastase [7]; the phthalide constituents (senkyunolide A and *Z*-ligustilide) of Ligusticum chuanxiong (Chuanxiong) inhibit TNF-α expression by reducing its mRNA stability [25] and exert anti-inflammatory effects by suppressing NF-κB and activating Nrf2 (Y. [28]). Similarly, a compound formula containing *Piper longum* (Biba) alleviates synovial hyperplasia, neovascularization, and cartilage destruction in the CIA model (Haiyue [38]); Angelica dahurica (Baizhi) possesses well-documented anti-inflammatory and analgesic properties (Hui [39]); the essential oil of *Cinnamomum tamala* (Guipi) exhibits anti-inflammatory activity, as validated by network pharmacology and experimental studies [23]. Collectively, these immunomodulatory and anti-inflammatory actions, delivered via transdermal absorption, may attenuate DTR-induced inflammatory responses and immune dysregulation, thereby reducing RA hospitalization risk.

### 4.4 Subgroup differences and seasonal variations

Stratified analyses revealed that the protective effect of SAA was more pronounced in vulnerable populations, including females, elderly individuals, and patients with multiple chronic comorbidities. The between-group difference in DTR effects was more pronounced in females, suggesting that women were both more susceptible to DTR exposure and more responsive to SAA therapy. Sex-based hormonal and physiological differences may account for this observation. Studies have shown that women generally have lower maximal oxygen consumption, which may make female patients with rheumatic diseases more susceptible to discomfort during rapid cooling [35]. Additionally, estrogen-related hormonal protection may contribute to women's better responsiveness to the active ingredients of Chinese materia medica delivered via SAA [22]. In males, the between-group difference was less pronounced, but as male patients constituted only 19.58% of the cohort (11.07% after PSM), this stratum had limited statistical power; the smaller observed difference in males should therefore be interpreted with caution rather than as evidence of no effect.

RA patients aged ≥45 years were more susceptible to the effects of DTR. Previous epidemiological evidence suggests that RA is associated with accelerated aging at midlife. Consequently, RA is more prevalent among middle-aged and elderly populations (aged ≥45 years), who are generally more prone to frequent and prolonged pain during weather changes [17]. Notably, SAA therapy exhibited a more pronounced therapeutic effect in this age group. This may be attributable to the increased rate of transdermal drug absorption in older individuals due to age-related reduction in skin barrier function [26]. Additionally, decreased metabolic clearance and reduced body water content in aging individuals may lead to higher plasma drug concentrations, thereby enhancing therapeutic efficacy [6].

We also observed a positive association between the number of chronic comorbidities and DTR-related RA hospitalization risk in the control group, but not in the SAA group. This suggests that SAA may provide greater protection for patients with a higher multimorbidity burden, possibly because these patients experience more pronounced baseline inflammation and immune imbalance that can be modulated by SAA [5].

Regarding seasonal variation, the protective effect of SAA was most pronounced in autumn, whereas no significant effect was observed in spring, summer, or winter. This is plausible because SAA is administered during the summer dog days and its protective effect may be strongest in the following autumn, when DTR variability is greatest and weather-related RA exacerbations are most frequent. In addition, low outdoor temperatures in winter may reduce people's outing times, thereby reducing their DTR exposure, which may explain the nonsignificant between-group difference in winter. In spring and summer, the relatively smaller DTR variability may likewise contribute to the absence of a significant between-group difference.

### 4.5 Clinical and public health implications

Our findings have implications for both clinical practice and public health policy regarding RA prevention and management. From a clinical perspective, routine RA treatments—which primarily focus on reducing disease activity and providing anti-inflammatory and analgesic effects—may not adequately prevent DTR-related disease exacerbation (J. [27]). This study establishes an epidemiological association between SAA therapy and a reduced risk of DTR-related RA hospitalization, with approximately 28% higher odds of DTR-related hospitalization in the control group than in the SAA group (*P* = 0.030). These findings underscore the necessity of integrating SAA into routine RA management, particularly for vulnerable populations including women, individuals aged ≥45 years, and those with multiple chronic comorbidities. Clinicians should inform RA patients of their DTR-related health risks, advise them to monitor sudden weather changes, and recommend SAA therapy during the summer dog days.

From a public health perspective, this study highlights the importance of incorporating complementary and alternative therapies—such as SAA—into health policy development. SAA may not only help control RA symptoms but also reduce the economic burden of the disease, which may encourage policymakers to develop and implement SAA-related disease prevention programs. Given that 304 hospitalizations in the control group after PSM were attributable to high DTR, even a partial offset of this burden would be economically meaningful. Furthermore, the association between DTR exposure and hospitalization risk can help alert RA patients and susceptible populations to adopt appropriate protective measures (e.g., SAA therapy, keeping warm, and reducing outdoor activities) to avoid exacerbation. Finally, our findings suggest that although meteorological factors are non-modifiable, proactive prevention can effectively mitigate meteorology-related disease risks. Policymakers should design more targeted proactive health strategies to promote public awareness of DTR-related disease prevention among vulnerable populations.

From a public health perspective, SAA therapy is feasible to scale. Firstly, as an external therapy, SAA's adverse effects are generally mild skin reactions [32], avoiding potential liver and kidney damage due to oral therapy. In addition, the SAA therapy protocol is standardized and can be administered by well-trained TCM physicians and centrally implemented in both TCM hospitals and community health centers. Secondly, SAA therapy is a low-cost, outpatient-based intervention that does not require hospitalization, expensive equipment, or long-term medication expenditures. More importantly, given that high DTR exposure is associated with 13.8% of RA hospitalizations in the control group, the promotion of SAA therapy may avert a considerable number of admissions and relieve the corresponding burden on the health insurance system. Finally, scaling SAA is consistent with the “Proactive Health” strategy advocated under the Healthy China initiative, which emphasizes the delivery of prevention-oriented disease interventions by hospitals and communities. Nevertheless, larger-scale randomized controlled trials and health economic evaluations must be conducted before nationwide scaling to confirm the efficacy and cost-effectiveness of SAA therapy.

### 4.6 Strengths and limitations

This study has several strengths. To our knowledge, it is the first to examine the modifying effect of SAA on DTR-related RA hospitalization risk, using a rigorous analytical framework that combines a time-stratified case-crossover design, propensity score matching, and attributable fraction estimation. The large sample size and long study period (12 years) provide sufficient statistical power for subgroup and sensitivity analyses.

Several limitations should also be noted. First, owing to incomplete residential address information for some patients, we used city-level meteorological data rather than individual-level exposures to measure DTR. Although we adopted a case-crossover design, which has been widely used in previous studies, potential exposure measurement error cannot be entirely eliminated. Second, the observational nature of this study precludes causal inference; future randomized controlled trials and mechanistic studies are needed to further validate these findings. Third, SAA therapy was provided on a voluntary, appointment-based basis rather than by randomization; patients who chose SAA may differ from controls in health awareness and healthcare-seeking behavior, and although PSM balanced age, sex, and number of comorbidities, residual selection bias cannot be completely excluded. Fourth, SAA-group hospitalizations accounted for only 12.57% of post-PSM records; the insignificant results in subgroup and seasonal analyses thus should be interpreted with caution. Finally, our analyses were based on data from only two tertiary TCM hospitals in Hefei, and we were unable to obtain data from other hospitals, so the findings may not be fully generalizable to regions with different climates, lifestyles, or healthcare systems. Moreover, the herbal cake used in SAA therapy was prepared by the hospital preparation Xiaoyu Jiegu powder, which is currently permitted only within these two hospitals and cannot yet be promoted to other cities, thereby limiting the external applicability of this intervention.

## 5 Conclusion

Based on the 12-year hospital data of 9261 RA patients, we found that high DTR exposure was associated with an increased risk of hospitalization. More importantly, we present the first evidence that SAA therapy was associated with a reduced risk of DTR-related RA hospitalization within one year. The relevance was more pronounced among women, elderly individuals, and patients with more comorbidities, and exhibited distinct seasonal variation—being most prominent in autumn. These findings provide novel insights into DTR-related RA risk reduction and suggest that proactive health interventions such as SAA may be beneficial for RA prevention. Policymakers and clinicians should consider integrating SAA into routine RA management and developing targeted proactive health services for vulnerable populations. Nevertheless, larger-scale randomized controlled trials and mechanistic research should be conducted to validate our results and provide a basis for incorporating SAA into RA management guidelines or preventive health strategies.

## CRediT authorship contribution statement

**Ling Xin:** Writing – original draft, Methodology, Investigation, Formal analysis, Conceptualization. **Jian Liu:** Supervision, Project administration, Methodology, Funding acquisition. **Wei Song:** Methodology, Data curation. **Yang Li:** Writing – original draft, Validation. **Yongjian Zhu:** Writing – review & editing, Methodology, Investigation.

## Ethics declaration

Written informed consent to take part in the study and to publish the article has been obtained from all participants or their legal representatives. The privacy rights of participants have been observed.

This study was performed in compliance with relevant laws, regulatory frameworks and guidelines where the research took place.

This study was approved by the Ethics Committee of the First Affiliated Hospital of Anhui University of Chinese Medicine.

(Approval No. 2025AH-57)

## Funding

This work was supported by the National Key Discipline of Chinese Medicine, Chinese Medicine Bi Disease (National Medical Education Letter [2023] No. 85), the National Chinese Medicine Inheritance and Innovation Project (Development and Reform Commission Social [2022] No. 366), the National Natural Science Foundation of China Project (82274490), and the Chinese Medicine Treatment Advantage Disease (Clinical Evidence Based Capacity Enhancement) Project (Wancaishe [2024] No. 1359).

## Declaration of competing interest

The authors declare that they have no known competing financial interests or personal relationships that could have appeared to influence the work reported in this paper.

## Data Availability

The datasets used and/or analysed during the current study are available from the corresponding author upon reasonable request.
