# Supplementary material for: Protective role of summer acupoint application therapy on weather-triggered rheumatoid arthritis flares
Source: One Health. 2026 Sep 14;23:101574. doi: 10.1016/j.onehlt.2026.101574 (PMC13594644; doi:10.1016/j.onehlt.2026.101574)
Supplement: Supplementary file 1 [file mmc1.docx]

Fig S1. Exposure-response curve for the associations between an IQR increase in DTR and hospitalisations for RA at a lag of 0-10 days

Fig S2. The association between an IQR increase in DTR and RA hospitalizations over 7 lag days, by gender, age, number of comorbidities, and seasons. Estimation results by adding the control variable, atmospheric pressure.

Fig S3. The association between an IQR increase in DTR and RA hospitalizations over 7 lag days, by gender, age, number of comorbidities, and seasons. Estimation results by adding the control variable, precipitation.

Fig S4. The association between an IQR increase in DTR and RA hospitalizations over 7 lag days, by gender, age, number of comorbidities, and seasons. Estimation results by adding the control variable, PM2.5.

Fig S5. The association between an IQR increase in DTR and RA hospitalizations over 7 lag days, by gender, age, number of comorbidities, and seasons. A natural cubic spline with 4 dfs was used to control meteorological variables.

Fig S6. The association between an IQR increase in DTR and RA hospitalizations over 7 lag days, by gender, age, number of comorbidities, and seasons. A natural cubic spline with 5 dfs was used to control meteorological variables.
