# Supplementary material for: Protective role of summer acupoint application therapy on weather-triggered rheumatoid arthritis flares
Source: One Health. 2026 Sep 14;23:101574. doi: 10.1016/j.onehlt.2026.101574 (PMC13594644; doi:10.1016/j.onehlt.2026.101574)
Supplement: Supplementary file 2 [file mmc2.docx]

Table S1. Spearman correlation coefficients between meteorological variables in Hefei, China, 2013–2024.

| Variables | DTR | TEM | RHU | WIN | NET |
| --- | --- | --- | --- | --- | --- |
| DTR | 1.000 |  |  |  |  |
| TEM | -0.011 | 1.000 |  |  |  |
| RHU | -0.641^**^ | 0.115^**^ | 1.000 |  |  |
| WIN | 0.001 | -0.046^**^ | 0.194^**^ | 1.000 |  |
| NET | -0.005 | 0.994^**^ | 0.087^**^ | -0.128^**^ | 1.000 |

Abbreviations: DTR Diurnal temperature range, TEM Mean temperature, RHU Relative humidity, WIN wind speed, NET Net effective temperature.

^*^ P<0.05, ^**^ P<0.01

Table S2. Baseline characteristics of the study population after PSM.

| Characteristic | Acupoint-stick group (N=1066) | Control group (N=1066) | P value |
| --- | --- | --- | --- |
| Gender |  |  |  |
| Female, n (%) | 946 (88.74%) | 950 (89.12%) | 0.783 |
| Male, n (%) | 120 (11.26%) | 116 (10.88%) | 0.783 |
| Age, year, Mean (SD) | 45.51 (12.79) | 45.39 (12.79) | 0.998 |
| Comorbidities |  |  |  |
| 0, n (%) | 801 (75.14%) | 805 (75.52%) | 0.841 |
| 1, n (%) | 140 (13.13%) | 140 (13.13%) | 1.000 |
| 2+, n (%) | 125 (11.73%) | 121 (11.35%) | 0.786 |
